# Kv1.1 null mice have enlarged hippocampus and ventral cortex

**DOI:** 10.1186/1471-2202-8-10

**Published:** 2007-01-24

**Authors:** Ann-Sophie Persson, Eric Westman, Fu-Hua Wang, Firoj Hossain Khan, Christian Spenger, Catharina Lavebratt

**Affiliations:** 1 Department of Molecular Medicine and Surgery, Karolinska Institutet, Stockholm, Sweden; 2 Department of Neurobiology, Health Care Sciences and Society, Karolinska Institutet, Stockholm, Sweden; 3 Department of Clinical Science, Intervention and Technology, Karolinska Institutet, Stockholm, Sweden; 4 Division of Disease Biology, AstraZeneca R&D Södertälje, Södertälje, Sweden; 5 Department of Physiology and Pharmacology, Karolinska Institutet, Stockholm, Sweden; 6 Section for Cellular and Genetic Therapy, Institute of Medical Microbiology, Rikshospitalet National Hospital, Oslo, Norway; 7 Department of Endocrinology, Diabetes and Medical Genetics, Medical University of South Carolina, Charleston, USA

## Abstract

**Background:**

Mutations in the *Shaker*-like voltage-gated potassium channel Kv1.1 are known to cause episodic ataxia type 1 and temporal lobe epilepsy. Mice that express a malfunctional, truncated Kv1.1 (BALB/cByJ-*Kv1.1*^*mceph*/*mceph*^) show a markedly enlarged hippocampus and ventral cortex in adulthood.

**Results:**

To determine if mice lacking Kv1.1 also develop a brain enlargement similar to *mceph/mceph*, we transferred Kv1.1 null alleles to the BALB/cByJ background. Hippocampus and ventral cortex was then studied using *in vivo *3D-magnetic resonance imaging and volume segmentation in adult Kv1.1 null mice, BALB/cByJ-*Kv1.1*^*mceph*/*mceph*^, BALB/cByJ-*Kv1.1*^*mceph*/+^, BALB.C3HeB -*Kv1.1*^-/+ ^and wild type littermates. The Kv1.1 null brains had dramatically enlarged hippocampus and ventral cortex. Mice heterozygous for either the null allele or the *mceph *allele had normal-sized hippocampus and ventral cortex.

**Conclusion:**

Total absence of Kv1.1 can induce excessive overgrowth of hippocampus and ventral cortex in mice with a BALB/cByJ background, while mice with one wild type Kv1.1 allele develop normal-sized brains.

## Background

The *megencephaly *BALB/cByJ-*Kv1.1*^*mceph*/*mceph *^mice (*mceph/mceph*), [1] show dramatic and progressive increase in brain size [2] after birth. The phenotype results from an 11 base-pair deletion in the gene encoding the *Shaker-*like voltage-gated potassium channel subunit Kv1.1 [3]. Kv1.1 is expressed in axons and dendrites of neurons and forms tetramers with other Kv1 subunits creating channels that regulate neuronal excitability and nerve signaling. The *mceph/mceph *mice express a truncated Kv1.1 protein in the endoplasmatic reticulum (ER), MCEPH, consisting of 230 out of 495 amino acids. MCEPH has a dominant negative effect on Kv1.2 and Kv1.3 currents when overexpressing MCEPH in *Xenopus *oocytes. However, in brain, no ER-retention of Kv1.2 was seen probably due to rapid MCEPH degradation and low MCEPH levels [4]. *mceph/mceph *mice behave normally at birth but from postnatal week 4 they begin to display progressive motor disturbances and typical behavior of complex partial seizures. The progressive brain overgrowth is significant at 8 weeks of age and affects primarily hippocampus and ventral cortex, whereas thalamus, olfactory bulb and cerebellum have normal size [2,5]. The enlarged areas have marked disturbances in expression of signaling components of brain derived neurotrophic factor (BDNF), Insulin like Growth factor (IGF) system, and several neuropeptides [3,5-7]. The hippocampal enlargement appears to result from both more and larger neurons and astrocytes [1,7]. BALB/cByJ mice heterozygous for the *mceph *mutation appear to behave normally but their brain has not been studied.

The link between this Kv1.1 truncation and brain overgrowth is not yet fully understood.

To establish if lack of functional Kv1.1 or the presence of the malfunctioning and truncated protein itself is the reason for the brain enlargement in *mceph/mceph*, we studied and compared hippocampus and ventral brain size using MRI in (1) animals with a complete lack of Kv1.1 (Kv1.1 null homozygotes), (2) animals that were lacking Kv1.1 in one allele but carried a functional Kv1.1 on the other allele (Kv1.1 null heterozygotes), (3) animals that had the truncating deletion in *Kv1.1 *in both (*Kv1.1*^*mceph*/*mceph*^, homozygotes) or only one allele (*mceph *heterozygotes). The wild type littermates of Kv1.1 null and *mceph/mceph *animals served as control groups. The *mceph *alleles and Kv1.1 null alleles were on genetic backgrounds of 100% and 94% BALB/cByJ, respectively. While the *mceph *mutation originated on BALB/cByJ, Kv1.1 null alleles were transferred through breeding from a C3HeB/FeJ background onto the background of theoretically 94% BALB/cByJ and 6% C3HeB/FeJ.

## Results and discussion

**Figure 1:**
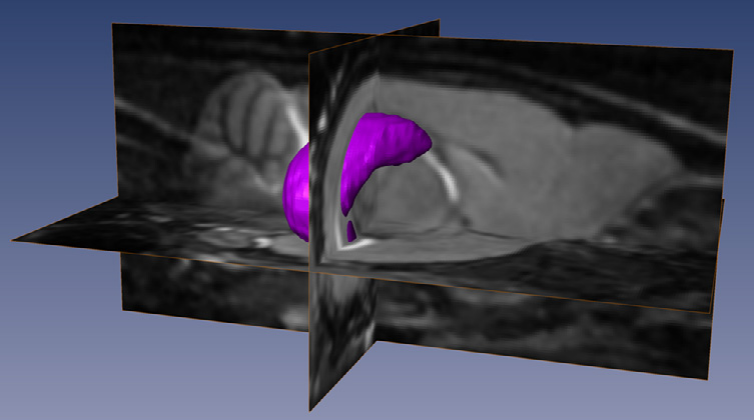
Reconstruction of the hippocampus within three planes from a 3D-MR image file. Segmentation and reconstruction was done using the Amira 3.0 software.

**Figure 2:**
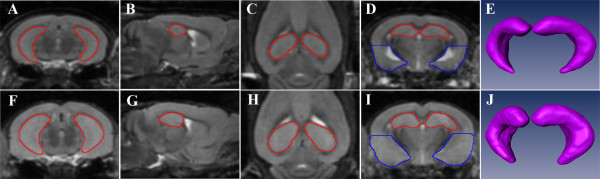
Segmentation of wild type and Kv1.1 null hippocampus and ventral cortex. The borders of the hippocampus (red) were drawn in three dimensions. Examples are shown for coronal (A, F), sagital (B, G) and horizontal (C, H) planes. The segmentation resulted in a 3D surface reconstruction of the hippocampus (E, J). The ventral cortex volume was derived from ventral cortex area (blue) measured in four coronal sections evenly distributed from 1.2 to 2.5 mm posterior to Bregma (D, I). Note the difference in size between wild type (top, i.e. A, B, C, D, E) and Kv1.1 null (bottom, i.e. F, G, H, I, J).

To elucidate whether knock-out of *Kv1.1 *can cause brain overgrowth as seen in the Kv1.1 truncated *mceph/mceph *mice, we first minimized the genomic variability between BALB/cByJ-*Kv1.1*^*mceph*/*mceph*^*(mceph/mceph) *and C3HeB/FeJ-Kv1.1 null mice by generating a semicongenic BALB.C3HeB-*Kv1.1*^-/- ^strain (Kv1.1 null). Brains from both *mceph/mceph *and Kv1.1 null mice as well as heterozygous and wild type littermates were then investigated for size of hippocampus and ventral cortex using 3D-MRI. For the hippocampus volume, 3D image files were used to manually segment the region and create a three dimensional view (Fig. 1). Examples of segmentation in all three orthogonal planes are shown in Figure 2. The ventral cortex was measured as depicted in Figure 2 (Fig. 2). Intra-rater and inter-rater reliability for the measurements of ventral cortex and hippocampus were in the range of *r *= 0.98–0.99.

**Figure 3:**
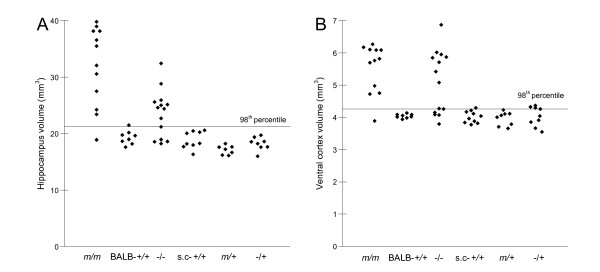
Hippocampus volume (A) and ventral cortex volume (B) in *Kv1.1 *mutants. Size values depict individual mice of the groups: BALB/cByJ-*Kv1.1*^*mceph*/*mceph *^(*m/m*), BALB/cByJ-*Kv1.1*^+/+^(BALB-+/+), semicongenic BALB.C3HeB -*Kv1.1*^-/- ^(-/-), BALB.C3HeB -*Kv1.1*^+/+ ^(s.c-+/+), BALB/cByJ-*Kv1.1*^*mceph*/+ ^(*m*/+) and BALB.C3HeB -*Kv1.1*^-/+ ^(-/+). The 98^th ^percentile of the hippocampus volume and ventral cortex volume from the +/+ (wild type) mice was set as arbitrary thresholds for enlargement.

**Figure 4:**
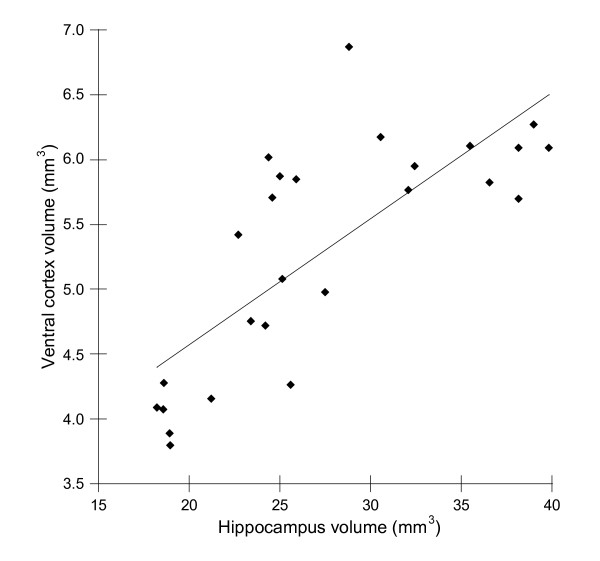
Correlation between hippocampus volume and ventral cortex volume. Size values from individual BALB/cByJ-*Kv1.1*^*mceph*/*mceph *^and semicongenic BALB.C3HeB-*Kv1.1*^-/- ^mice are given. The regression line is shown and the correlation is very good (*r *= 0.76, 95% CI: 0.53–0.89).

Both pure BALB/cByJ wild type and semicongenic wild type (94% BALB/cByJ and 6% C3HeB/FeJ) littermates were used as controls. The mice of these two control groups were very similar, that is no significant difference was observed in hippocampus volume (Mean ± SD: 19.3 ± 1.2 mm^3 ^for BALB/cByJ; 18.9 ± 1.5 mm^3 ^for semicongenic) or ventral cortex volume (4.04 ± 0.06 mm^3 ^for BALB/cByJ; 4.01 ± 0.18 mm^3 ^for semicongenic) between these two groups (Fig. 3). Comparing *mceph/mceph *mice with age-matched BALB/cByJ wild type mice showed that *mceph/mceph *mice had a 66% larger hippocampus (32.0 ± 7.0 mm^3^, *t *= 6.1, 18 *df*, *P *= 0.0001) and a 37% larger ventral cortex (5.53 ± 0.76 mm^3^, *t *= 6.8, 18 *df, P *= 0.00001). Eleven of the 12 *mceph/mceph *brains had a hippocampal volume and a ventral cortex volume greater than any wild type littermate. Likewise, 11 of the 12 *mceph/mceph *animals had hippocampus and ventral cortex enlargement above the 98^th ^percentile of the wild type mice (Fig. 3). Similarly to *mceph/mceph*, several of the Kv1.1 null brains showed a clearly enlarged hippocampus and ventral cortex. However, enlargement was found in a smaller percentage in Kv1.1 null than in *mceph/mceph *mice (69% in Kv1.1 null versus 92% in *mceph/mceph*). Nevertheless, we could demonstrate that the lack of Kv1.1 can result in an enlargement of both hippocampus and ventral cortex (Fig. 2). Of the 13 analyzed Kv1.1 null brains, 9 had a hippocampal volume above, and 8 of these 9 had a ventral cortex volume above the 98^th ^percentile of wild type mice (Fig. 3). Kv1.1 null heterozygotes, carrying only one Kv1.1 allele, showed no significant enlargement of hippocampus (18.2 ± 1.2 mm^3^) or ventral cortex (4.03 ± 0.30 mm^3^). Similarly, the 8 *mceph *heterozygotes showed a hippocampus volume (17.1 ± 0.80 mm^3^) or ventral cortex volume (3.96 ± 0.21 mm^3^) comparable to their wild type littermates (Fig. 3). The hippocampal volume and the ventral cortex volume correlated very well for the *mceph/mceph *and the Kv1.1 null mice (*r *= 0.76, 95% CI: 0.53–0.89) (Fig. 4).

Behavior and appearance of the Kv1.1 null mice were similar to that of the *mceph/mceph *mice and markedly different from that of the wild type littermates. The phenotypes include body tremor, a hyperstartle response, jittering, forelimb paddling and cramps, a sitting posture, audio sensitivity, porphyria, as well as inflammation and crusting of eye lid and the outer ear. These pathologic signs were present in all *mceph/mceph *and all Kv1.1 null mice, but not in heterozygous or wild type mouse. Kv1.1 mice had a milder epileptic behavior than *mceph/mceph *mice. Nine of 9 *mceph/mceph *mice had class IV, and 7 of 9 *mceph/mceph *mice had class V seizures while only 4 out of the 6 Kv1.1 null mice with enlarged brain regions had class IV seizures and 2 of these 4 Kv1.1 null mice had class V seizures. The 3 Kv1.1 null mice with normal-sized brains had no class IV or V seizures.

Hence, lack of Kv1.1 can cause a significant enlargement of ventral cortex and hippocampus, while one Kv1.1 allele was enough for normal brain region size. The variability in brain size was larger among the Kv1.1 null mice than among the *mceph/mceph *mice (6 of 13 Kv1.1 compared to 1 of 12 *mceph/mceph *mice had brain sizes within the wild type range). A likely explanation for this is the presence of 6% C3HeB/FeJ genomic background in the Kv1.1 null mice, whereas the *mceph/mceph *mice have a pure BALB/cByJ background. We know that the *mceph *trait has reduced penetrance in intercrosses to other strains [3]. The Kv1.1 null mice are epileptic on all genetic backgrounds tested including 129/SvJ, N:NIH-BC, C57BL/6J, C3HeB/FeJ, Swiss Black and hybrids thereof, but has not previously been reported to have excessive brain growth [8]. Investigation of brain morphology in 3 months old Kv1.1 null mice on 129/SvJ × Swiss black hybrid background revealed only the typical effects of seizures in the hippocampus: neuronal loss and mossy fiber sprouting [9]. Hence, we here report for the first time that Kv1.1.null mice can display excessive brain growth. A factor possibly increasing the severity of the brain overgrowth and the seizure behavior in *mceph/mceph *mice is heteromultimerization with, and hence retention of, other Kv1 subunits in ER by the MCEPH protein expressed in *mceph/mceph *mice [4]. We can only speculate about how lack of Kv1.1 and overgrowth of hippocampus and ventral cortex could be linked on BALB/cByJ and what factors that modify this link on other genetic backgrounds. One possible explanation would be seizure-induced upregulation of trophic factors affecting cellular growth, proliferation and survival. This is supported by our observation that enlargement and elevated levels of trophic factors co-occurred spatially in *mceph/mceph *brain [2]. Also, the *mceph/mceph *mice display, at least in hippocampal dentate gyrus, increased proliferation, neurogenesis and enhanced cell survival, as well as more neurons and astrocytes in several hippocampal regions [10]. It is known that there are marked differences between mouse strains in cell proliferation as well as survival rate of the new cells in the hippocampus under normal conditions [11]. Also, seizures are known to induce cell proliferation and apoptosis [12,13], with the intensity of seizures being of importance for the balance between generation of neurons and neuronal loss [14]. Importantly, there are significant differences between mouse strains in the sensitivity and resistance of neurons to seizures [15,16]. Thus, the different steps linking lack of Kv1.1 with brain overgrowth need to be further elucidated. Nevertheless, the results in this study propose the possibility that some patients with epilepsy and enlarged brain (megalencephaly) might suffer from lack of functional Kv1.1 or related protein. Hence, screening for mutations in the genes encoding Kv1 subunits should maybe be considered for these patients.

## Conclusion

We were here able to demonstrate that absence of Kv1.1 can lead to an increased growth of the brain. Also, the genetic background is critical and the BALB/c is suitable for this study.

## Methods

### Mice

The BALB/cByJ-*Kv1.1*^*mceph*/*mceph *^(*mceph/mceph*), BALB/cByJ-*Kv1.1*^+/+ ^(wild type) and C3HeB/FeJ-*Kcna1*^tm1Tem ^(C3HeB/FeJ-*Kv1.1*^-/-^, Kv1.1 null). (Smart et al., 1998) mouse strains were obtained from The Jackson Laboratory (Bar Harbor, ME, USA). The mice were kept in a barrier animal facility at 12 h light: 12 h darkness, a temperature of 21–22°C and a relative humidity of 40–50%. Rodent breeding diet R70 (Lactamin AB, Stockholm, Sweden) and water were provided *ad libitum*. All studies were approved by the Northern Stockholm Ethical Committee on Experimental Animal Care and performed in accordance with guidelines from the Swedish National Board for Laboratory Animals.

### Generation of a BALB.C3HeB-Kv1.1^-/- ^semicongenic strain

C3HeB/FeJ-*Kv1.1*^-/- ^mice were outcrossed to BALB/cByJ-*Kv1.1*^+/+^. F1 males/females heterozygous for the Kv1.1 null locus were backcrossed to the recipient strain BALB/cByJ-*Kv1.1*^+/+^. Heterozygous mice from the N4 generation were mated to give homozygous Kv1.1 null offspring. These Kv1.1 null mice (((C3HeB/FeJ-*Kv1.1*^-/- ^× BALB/cByJ-*Kv1.1*^+/+^) × BALB/cByJ-*Kv1.1*^+/+^) N4F2) as well as their Kv1.1 null heterozygous and wild type littermates were studied.

### PCR-based genotyping

Presence of the *mceph *mutation was determined through amplification of a fragment covering the 11 base pair deletion. The PCR products were size separated on 3% agarose gels and visually inspected for determination of genotype. Presence of the Kv1.1 null allele was determined using the NEOTD protocol provided by The Jackson Laboratory. This protocol can not distinguish between homozygotes and heterozygotes so this was done by checking for the presence of a *Kv1.1 *gene fragment.

### 3-dimensional Magnetic Resonance Imaging (3D-MRI)

Mice studied were 12 weeks old and include 12 BALB/cByJ-*Kv1.1*^*mceph*/*mceph *^(*mceph/mceph*), 8 BALB/cByJ-*Kv1.1*^+/+ ^(wild type), 8 BALB/cByJ-*Kv1.1*^*mceph*/+ ^(*mceph *heterozygots), 13 semicongenic BALB.C3HeB-*Kv1.1*^-/- ^(Kv1.1 null), 10 semicongenic BALB.C3HeB-*Kv1.1*^+/+ ^(wild type), and 9 semicongenic BALB.C3HeB-*Kv1.1*^-/+^(Kv1.1 null heterozygots). For MRI, mice were anesthetized with 1.5–2.0 % isofluran in air delivered via a mouthpiece allowing spontaneous respiration. The mouse was positioned in supine position on an acrylic cradle and the head was placed in the center of the coil. To reduce motion artifacts, the head of the mouse was fixed to the rig. Body temperature was measured with a rectal probe and maintained at 36 to 37°C using an MRI-compatible air temperature control system.

MRI was performed using a 4.7 T, 40 mm bore horizontal magnet (Bruker Biospec Avance 47/40, Bruker, Karlsruhe, Germany) fitted with a 12 cm inner diameter self-shielded gradient system (max. gradient strength 200 mT/m). A volume coil (Bruker) with 25 mm inner diameter was used for excitation and signal detection. The sequence employed for 3D-imaging was an inversion recovery (IR) spin echo sequence with rapid acquisition with relaxation enhancement (RARE) imaging [17]. The sequence was optimized for maximum contrast of the hippocampus with parameters: repetition time (TR) 2566.8 ms, echo time (TE) 35.6 ms, RARE-factor 8 with RARE-maximum 4, inversion time 450 ms, matrix size 64 × 64 × 128 and 2 averages. Field of view (FOV) for the 3D was 0.90 × 1.20 × 1.80 cm resulting in a resolution of 0.14 × 0.18 × 0.14 mm in dorso-ventral, left-right and rostro-caudal directions, respectively.

3D-MR images were analyzed by using Amira 3.0 software (Mercury Computer Systems, GmbH). The border of the hippocampus was drawn at the border with the fimbria and corpus callosum and checked in three orthogonal planes to ensure accuracy. Measurements of ventral cortex area were performed as previously detailed [2] in four coronal sections spanning the interval between 1.2 and 2.5 mm posterior to Bregma (every second or third consecutive section, [18]) using Bruker standard software (Paravision 3.0.2). The rhinal fissure defined the dorsal boundary of the ventral cortex [2] and the dark fimbria defined the medial boundary of the ventral cortex. The amygdala was included in the region defined as ventral cortex. Measurements were performed twice and the mean value calculated. Ventral cortex volume was expressed as measured area (mm^2^) × slice thickness and presented for one hemisphere. Intra-rater reliability was assessed by measuring right ventral cortex area ten times in 10 *mceph/mceph *mice, and by measuring the hippocampus volume twice in five mice. For the ventral cortex, the average correlation coefficient (*r*) between the repeated measurements was 0.99. The mean ventral cortex area of the 10 mice was then compared between any two repeated measurements. The mean and SEM of the difference between any two repeated measurements was 2.9% and 2.3%, respectively. For the hippocampus volume, mean and SEM of the set of five mice differed between the two repeated measurements with 5.9% and 15.2%, respectively, and *r *was 0.99 between repeated measurements. Inter-rater reliability was assessed by calculating *r *between the measurements of two independent investigators as described previously [2] and included measurements of ventral cortex volume from five *mceph/mceph *and five wild type mice. The mean and SEM of measured volumes differed between investigators with 3.1% and 13.8%, respectively, and the correlation coefficientbetween the investigators was *r *= 0.98.

### Seizure assessment

Presence of class IV and V epileptiform activities (according to classification by Racine [19]) was determined in 9 *mceph/mceph *and 9 Kv1.1 null mice aged 11–12 weeks by visual scoring of records from video camera (SSC-M183CF, Sony Corporation, Tokyo, Japan), and time lapse security recorder (HS-1024, Mitsubishi Electric, Tokyo, Japan). Class IV is defined as rearing, and class V as rearing followed by loss of postural control and falling. These classes are considered as models of generalized complex partial seizures. Class V is considered a full motor seizure. The mice were videotaped during both the night and the day for 3 h each, starting 3 h before and 6 h after the turn off of light.

### Statistical analysis

Sizes of brain regions were compared using two-tailed non-paired *t*-tests, after checking for equality in variance in the compared groups using Bartlett's test. The null hypothesis was rejected if *P *< 0.017 (Bonferroni correction for 4 comparisons). The statistical analyses were performed using STATA version 8.2 (Stata Corporation, College Station, Texas).

## Abbreviations

MRI, magnetic resonance imaging

## Authors' contributions

ASP carried out genotyping, the volume assessment, and drafted the manuscript. EW and FHW carried out the MRI experiments and measured region areas. FHK participated in generating and genotyping the mice. CS coordinated the MRI experiments, participated in MRI protocol design and was the main critical reviser of the manuscript. CL conceived and coordinated the study, organized the generation of the mice, measured region areas, performed statistical analysis and participated in drafting and revising the manuscript. All authors read and approved the final manuscript.
